# End-Edge-Cloud Collaborative Monitoring System with an Intelligent Multi-Parameter Sensor for Impact Anomaly Detection in GIL Pipelines

**DOI:** 10.3390/s26020606

**Published:** 2026-01-16

**Authors:** Qi Li, Kun Zeng, Yaojun Zhou, Xiongyao Xie, Genji Tang

**Affiliations:** 1 Department of Geotechnical Engineering, School of Civil Engineering, Tongji University, Shanghai 200092, China; 2310026@tongji.edu.cn (Q.L.); xiexiongyao@tongji.edu.cn (X.X.); 2013tanggenji@tongji.edu.cn (G.T.); 2 Key Laboratory of Geotechnical and Underground Engineering of Ministry of Education, School of Civil Engineering, Tongji University, Shanghai 200092, China; 3 State Grid Shanghai Municipal Electric Power Company, Shanghai 200072, China; zyjwnl600@163.com

**Keywords:** gas-insulated transmission line, structural health monitoring, end-edge-cloud monitoring, impact-location identification, extreme gradient boosting, intelligent multi-parameter sensor

## Abstract

Gas-insulated transmission lines (GILs) are increasingly deployed in dense urban power networks, where complex construction activities may introduce external mechanical impacts and pose risks to pipeline structural integrity. However, existing GIL monitoring approaches mainly emphasize electrical and gas-state parameters, while lightweight solutions capable of rapidly detecting and localizing impact-induced structural anomalies remain limited. To address this gap, this paper proposes an intelligent end-edge-cloud monitoring system for impact anomaly detection in GIL pipelines. Numerical simulations are first conducted to analyze the dynamic response characteristics of the pipeline under impacts of varying magnitudes, orientations, and locations, revealing the relationship between impact scenarios and vibration mode evolution. An end-tier multi-parameter intelligent sensor is then developed, integrating triaxial acceleration and angular velocity measurement with embedded lightweight computing. Laboratory impact experiments are performed to acquire sensor data, which are used to train and validate a multi-class extreme gradient boosting (XGBoost) model deployed at the edge tier for accurate impact-location identification. Results show that, even with a single sensor positioned at the pipeline midpoint, fusing acceleration and angular velocity features enables reliable discrimination of impact regions. Finally, a lightweight cloud platform is implemented for visualizing structural responses and environmental parameters with downsampled edge-side data. The proposed system achieves rapid sensor-level anomaly detection, precise edge-level localization, and unified cloud-level monitoring, offering a low-cost and easily deployable solution for GIL structural health assessment.

## 1. Introduction

Gas-insulated metal-enclosed transmission lines (GILs) are high- and ultra-high-voltage systems that employ a metal-enclosed coaxial structure filled with an SF_6_/N_2_ gas mixture or emerging low-global-warming-potential insulating gases [1,2]. Each GIL pipeline typically consists of an inner conductor, metal enclosure, support insulators, and flange joints [3]. With their high dielectric strength, low electromagnetic emissions, large current-carrying capacity, and excellent environmental adaptability, GIL pipelines are widely deployed in underground or partially enclosed installations where safety and space are strictly constrained [4]. Common applications include river-crossing and undersea tunnels, urban utility tunnels, subway sections, and large substations. GIL pipelines are increasingly becoming essential components of modern high-density urban power transmission networks [5,6].

With the continuous growth of electrical load in urban cores and the increasing scarcity of available land, many older substations in central urban areas now face the need for expansion and renovation. Under such conditions, construction while in operation has become commonplace. In this context, external disturbances—such as pile foundation work, shield tunneling, and the hoisting or transportation of heavy equipment in adjacent civil engineering projects—can induce mechanical impacts, vibration disturbances, and even localized structural damage to operating GIL pipelines [6,7]. Consequently, real-time and reliable monitoring of the operational status and structural integrity of GIL pipelines in complex urban construction environments has become a critical requirement to ensure the safe operation of urban power grids and to support substation expansion projects.

The current GIL operation monitoring systems primarily focus on three categories: electrical insulation condition, internal gas environment parameters, and structural dynamic response. Regarding electrical insulation monitoring, partial discharge (PD) has long been recognized as a key indicator for assessing insulation degradation and detecting early defects [8]. To effectively detect PD events, ultra-high-frequency (UHF) sensors are commonly employed to capture discharge electromagnetic pulse signals, enabling high-sensitivity identification and localization of internal electrical defects [9]. Jiang et al. [10] systematically evaluated conventional time-difference-of-arrival (TDoA) extraction methods—including cross-correlation function (CCF), minimum energy (ME), and threshold value (TV) approaches—for UHF PD signals, and proposed a combined averaging strategy that fuses multiple algorithms to reduce TDoA estimation error to below 4%, thereby improving PD source localization accuracy under field conditions. Khodaveisi et al. [11] achieved high-accuracy three-dimensional localization of PD sources inside power transformer tanks using a single electric field sensor in combination with multiple machine learning models. Khan et al. [12] proposed an efficient algorithm based on received signal strength (RSS) for PD localization in high-voltage substations, employing a UHF sensor array to enable low-cost asynchronous source positioning while mitigating the adverse effects of uncertain path-loss model parameters.

Apart from electrical defects, the gas temperature, pressure, and flow distribution inside GIL pipelines also directly influence insulation lifespan and the likelihood of PD [13,14]. Consequently, monitoring temperature rise and gas conditions is an essential aspect of operational safety [15,16]. Wang et al. [17] observed that a greater increase in SO_2_F_2_ compared to SO_2_ serves as an indicator of arc discharge, which was attributed to mechanical looseness through online monitoring of SF_6_ decomposition byproducts. Li et al. [18] reported that as temperature rises, the number of PD events increases significantly while the PD inception voltage (PDIV) decreases markedly, suggesting that elevated temperatures accelerate electrical treeing and enhance the probability of discharge activity. Ma et al. [19] employed deep learning to reconstruct high-resolution gas concentration fields from sparse sensor measurements, enabling the generation of comprehensive gas-state maps and providing a novel data-driven approach for accurately monitoring key physical parameters within GIL pipelines.

The structural response of GIL pipelines is another critical indicator directly related to operational safety. Existing research primarily focuses on finite element-based dynamic analysis and structural health monitoring using vibration responses [20]. Li et al. [21] constructed a three-dimensional finite element model of a 550 kV GIL pipeline. Through modal analysis and response spectrum analysis, they evaluated the vibration modes, natural frequencies, and stress distributions under seismic loading, verified the overall seismic resilience of the structure, and identified the support bracket as a structural weak point. Li et al. [22] developed a refined finite element model of an overhead GIL pipeline that accounts for internal conductor sliding and joint clearance, revealing the pipeline height-change corner as the seismic vulnerability with two failure modes: housing strength failure and excessive conductor displacement. They proposed a “fully fixed supports” retrofit strategy that reduces the probability of seismic failure by over 50%. Zhang et al. [23] addressed the operation and maintenance challenges of long-distance and high-drop GIL pipelines by proposing a multi-parameter integrated monitoring scheme based on distributed optical fibers, enabling synchronous measurement of temperature, strain, and vibration. Wang et al. [24] established a 110 kV GIL pipeline test platform, simulated three typical mechanical faults—screw loosening, external impacts, and incomplete embedding of the conductive rod—and collected vibration acceleration signals from the pipeline shell. Mechanical fault modes were identified and diagnosed using a combined method of fuzzy entropy applied to complementary ensemble empirical mode decomposition (CEEMD) and the whale optimization extreme learning machine (WOA-ELM) model. Jiang et al. [25] employed wavelet packet decomposition and principal component analysis (PCA) for dimensionality reduction in low-frequency vibration signals, constructing a support vector machine (SVM) model to achieve high-accuracy classification of three states: normal, insulator damage, and loose solder joints. Xu et al. [26] integrated acoustic and vibration signals, proposed a joint acoustic-vibration positioning method, and enhanced traditional SVM performance using the seagull optimization algorithm (SOA). In addition, several studies have investigated impact detection on GIL or similar pipeline structures based on vibration responses, using distributed acoustic sensing (DAS) [27,28] and acoustic emission (AE) [29,30] techniques to capture transient vibration signals induced by sudden external impacts. Overall, current GIL structural response monitoring research remains largely centered on extracting features from vibration or acoustic signals and performing fault or state identification. However, lightweight and integrated solutions capable of providing rapid, on-edge early warning for GIL pipelines are still lacking.

This paper presents an end-edge-cloud monitoring architecture designed to address potential structural impact anomalies that GIL pipelines may experience during substation expansion, equipment hoisting, and other construction activities. These impact anomalies are detected and analyzed based on the vibration responses of the pipeline, with the collected acceleration and angular velocity signals serving as the foundation for signal processing and technical diagnosis. At the end tier, an intelligent multi-parameter sensor is developed to enable rapid on-site detection of impact events. At the edge tier, an impact-location recognition model is trained on a personal computer (PC) using data collected from laboratory impact tests. At the cloud tier, a lightweight visualization platform is implemented to support integrated monitoring of structural responses and environmental parameters. Laboratory results show that the proposed system achieves impact detection at the sensor level, accurate impact localization at the edge, and real-time visualization in the cloud, enabling multi-layer collaborative monitoring of impact anomalies in GIL pipeline structures.

The remainder of this paper is organized as follows. Section 2 introduces the principle of the impact-location identification algorithm trained on the PC side and describes the overall architecture of the end-edge-cloud monitoring framework. Section 3 analyzes the structural response mechanisms of the GIL pipeline under various impact cases through numerical simulations. Section 4 describes the hardware design and functionality of the developed intelligent multi-parameter sensor. Section 5 presents the laboratory experiments, including sensor data acquisition, model training and validation for impact-location identification, and cloud-based visualization of the monitoring results. Section 6 concludes the paper.

## 2. Methodology and System Architecture

### 2.1. Extreme Gradient Boosting

XGBoost (extreme gradient boosting) is an efficient ensemble learning algorithm built upon the gradient boosting decision tree (GBDT) framework, originally proposed by Chen and Guestrin in 2016 [31]. Compared with the traditional GBDT approach, XGBoost incorporates comprehensive improvements in objective function formulation, regularization, node-splitting strategies, and system-level optimization, thereby substantially enhancing both model expressiveness and computational efficiency [32]. Regarding the optimization objective, XGBoost approximates the overall objective function by performing a second-order Taylor expansion of the loss function. At the same time, it incorporates the first-order gradient term, the second-order gradient term, and a regularization term controlling model complexity into the objective function. This design improves the accuracy of the optimization direction while mitigating overfitting. The general form of the objective function can be expressed as

(1) $$Obj=\sum_{i=1}^{n}l(y_{i},{\hat{y}}_{i})+\sum_{k=1}^{K}\left(\gamma T_{k}+\frac{1}{2}\lambda \sum_{j=1}^{T_{k}}w_{kj}^{2}\right)$$

where $l(y_{i},{\hat{y}}_{i})$ denotes the loss term, $\sum_{k=1}^{K}\left(\gamma T_{k}+\frac{1}{2}\lambda \sum_{j=1}^{T_{k}}w_{kj}^{2}\right)$ represents the regularization term, γ is the penalty coefficient for the number of leaf nodes, λ indicates the regularization strength of the leaf node weights, with $T_{k}$ and $w_{kj}$ correspond to the number of leaf nodes and their associated weights in the k-th tree, respectively.

Regarding feature processing, XGBoost can automatically handle missing values and, through a sparse-aware splitting strategy, identify the optimal split direction even when features are missing or contain zero values. The algorithm also incorporates techniques such as column sampling, learning rate reduction, and post-pruning, which collectively improve the model’s generalization capability [33]. From a system optimization perspective, XGBoost leverages cache-aware candidate split computation, feature-level and data-level parallelism, as well as a distributed training framework, making it well-suited for high-dimensional feature inputs and complex classification tasks [34,35]. Owing to these advantages, XGBoost has been widely applied in areas including structural health monitoring, mechanical fault diagnosis, and multi-source signal analysis, demonstrating strong robustness and predictive performance [36,37].

### 2.2. End-Edge-Cloud Monitoring Architecture

**Figure 1:**
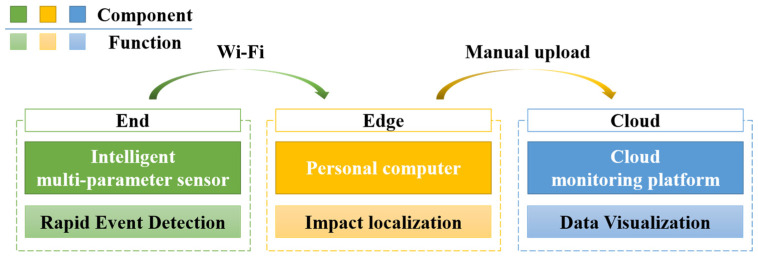
End-edge-cloud monitoring architecture.

This paper presents a lightweight, three-tiered end-edge-cloud monitoring system for detecting potential impact anomalies in the routine operation and maintenance of GIL pipelines. The system establishes a complete monitoring chain encompassing multi-source data acquisition, rapid event detection, impact location identification, and historical data management. Designed following engineering principles, it emphasizes a non-intrusive approach with low power consumption and ease of installation, making it primarily suitable for GIL pipelines in large-scale substations while also being applicable to pipelines in tunnels and utility corridors. As shown in Figure 1, the system consists of the intelligent multi-parameter sensor (end), the edge computing PC (edge), and the cloud-based visualization platform (cloud), with each module collaboratively performing detection, localization, and recording of impact events.

The intelligent multi-parameter sensor on the end side is responsible for original data collection and rapid event detection. The sensor can simultaneously acquire multi-source data, including triaxial acceleration, triaxial angular velocity, temperature, humidity, and strain and performs preprocessing at the sensor level to achieve data denoising, feature extraction, and threshold-based event detection. It operates in two modes: continuous acquisition and event-based acquisition. In the event-based mode, once an impact signal exceeds the threshold, only the key data from the event point and a defined time window are uploaded to the PC via Wi-Fi, thereby reducing data transmission volume.

The edge PC is primarily responsible for intelligent impact location recognition. Based on laboratory test data, an XGBoost multi-class model is constructed using six features—triaxial acceleration and triaxial angular velocity—to enable accurate identification of different impact positions. Upon receiving event data uploaded from the sensor, the PC performs impact location recognition and stores both the original signals and the recognition results locally for subsequent historical record management and cloud visualization. In the current system design, the cloud platform relies on manual data reporting: after the PC completes the recognition, the user can choose whether to upload the data to the cloud to ensure that only valid monitoring data are displayed.

The cloud platform functions as the central hub for information display and historical record management within the monitoring system, providing visualizations of the monitoring data and impact identification results uploaded from the PC. The platform’s homepage presents the operational environmental parameters of the pipeline, while users can access detailed historical records for each sensor, including triaxial acceleration, triaxial angular velocity, and recorded impact events.

In practical applications, when an impact event occurs, the intelligent multi-parameter sensor rapidly detects the anomaly by monitoring sudden changes in the peak value of the X-direction acceleration, while simultaneously collecting three-axis acceleration and angular velocity data at the monitoring point. In event-based acquisition mode, the sensor transmits the structural dynamic response data from a defined time window before and after the impact to the edge PC. The edge PC then employs the pre-trained XGBoost model to identify the impact location and stores both the raw data and recognition results locally. Users can subsequently choose to manually upload the data to the cloud platform for historical visualization and further analysis.

## 3. Numerical Study of GIL Structural Response

### 3.1. Model Development and Load Application

**Figure 2:**
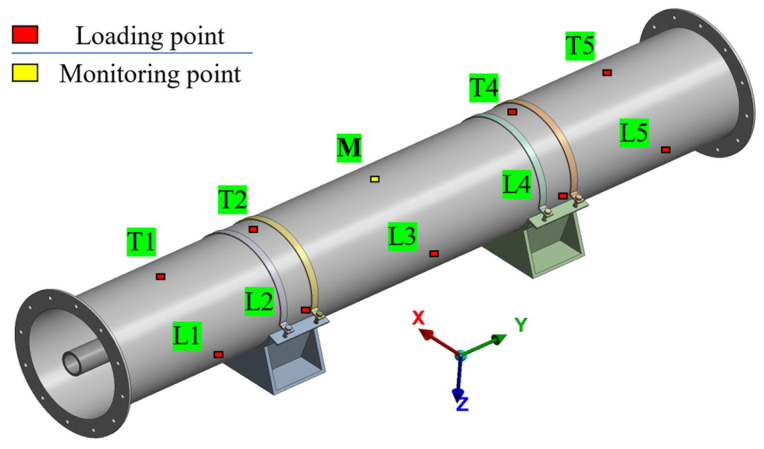
Pipeline model and load application locations.

To investigate the effects of abnormal impacts at different locations and magnitudes on the structural response of the GIL pipeline, a numerical model of a GIL pipe section is first developed. The acceleration response at the top midpoint of the section (point M in Figure 2) is analyzed under various loading cases, and this point is designated as the subsequent monitoring location.

The pipe section model consists of a shell, three-post insulators, a central guide rod, clamps, and a base. The shell has a length of 6 m and a diameter of 0.9 m, and it is secured by clamps and a base fastened with bolts. The shell and central guide rod are made of aluminum alloy, the three-post insulators are made of epoxy resin, and the clamps and base are made of stainless steel.

As shown in Figure 2, five sections along the Y-axis are selected, and impact loads of varying magnitudes are applied to both the top and lateral points of the shell at each section. In total, nine loading points are considered, while the top point of the middle section is designated as the monitoring point and is not subjected to any external loading. The total simulation duration is 1 s. The impact loads are modeled as symmetrical triangular pulses with a duration of 10 ms, applied between 0.495 s and 0.505 s, reaching the peak at 0.5 s, with magnitudes of 500 N, 1000 N, and 2000 N, respectively. In total, 27 loading cases are simulated. The designations of these cases are provided in Table 1 and Table 2. Using T1-500 as an example, T1 denotes the location of the loading point, and 500 indicates that the peak magnitude of the impact force is 500 N.

### 3.2. Pipeline Structural Responses Under Different Loads

**Figure 3:**
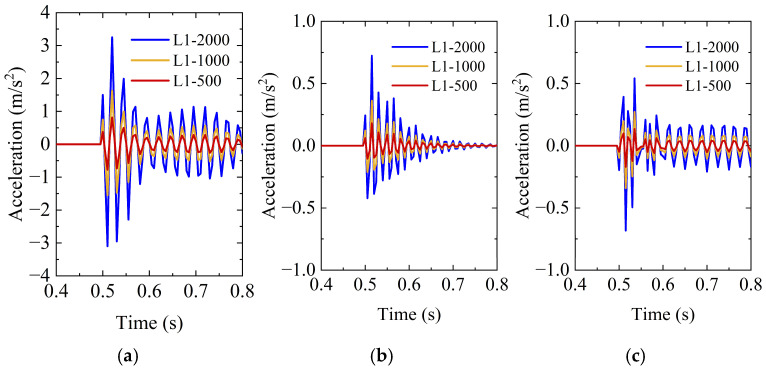
Structural responses under different load magnitudes: (**a**) MX; (**b**) MY; and (**c**) MZ.

As shown in Figure 3, when loads of different magnitudes are applied at the same location, the overall pattern of the acceleration response at the measurement point remains consistent. Here, MX denotes the acceleration response of the monitoring point M in the X direction. Taking the X-direction acceleration as an example, the peak values under load magnitudes of 500 N, 1000 N, and 2000 N are 0.813, 1.626, and 3.251 m/s^2^, respectively. The peak acceleration shows a strong positive correlation with the applied load.

**Figure 4:**
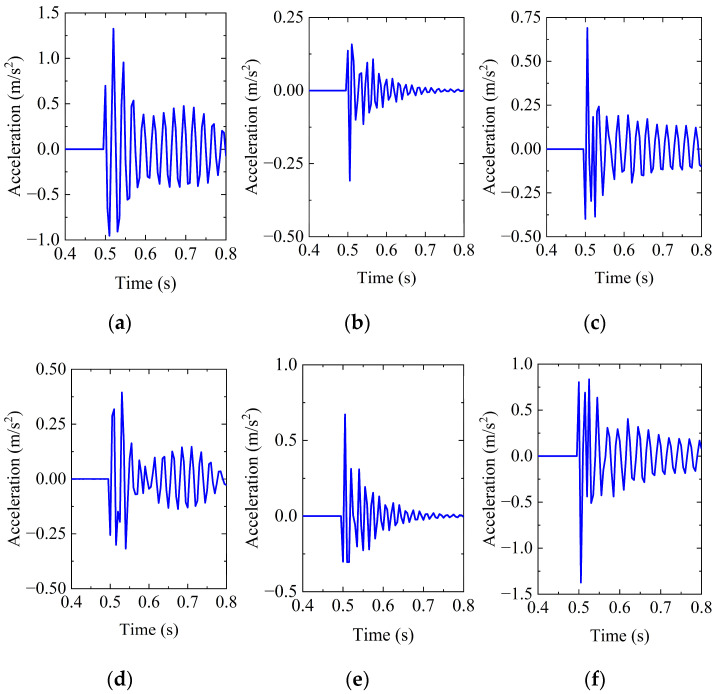
Structural responses under different load directions: (**a**) MX(L2-1000); (**b**) MY(L2-1000); (**c**) MZ(L2-1000); (**d**) MX(T2-1000); (**e**) MY(T2-1000); and (**f**) MZ(T21000).

As shown in Figure 4, when a lateral load is applied, the pipeline primarily generates lateral vibration, causing the peak acceleration at the measurement point in the X direction to be the largest. When a load is applied at the top, the pipeline primarily generates vertical vibration, causing the peak acceleration in the Z direction to be the largest. The direction of the maximum peak acceleration aligns with the direction of the applied load, indicating that the acceleration response of the pipeline under impact loading exhibits clear directional selectivity. Here, MX(L2-1000) represents the X-direction acceleration response at the monitoring point M resulting from an impact load with a peak magnitude of 1000 N applied at L2, with other responses labeled in the same way.

**Figure 5:**
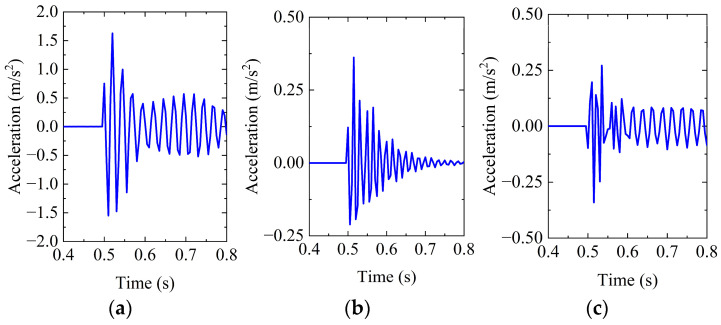

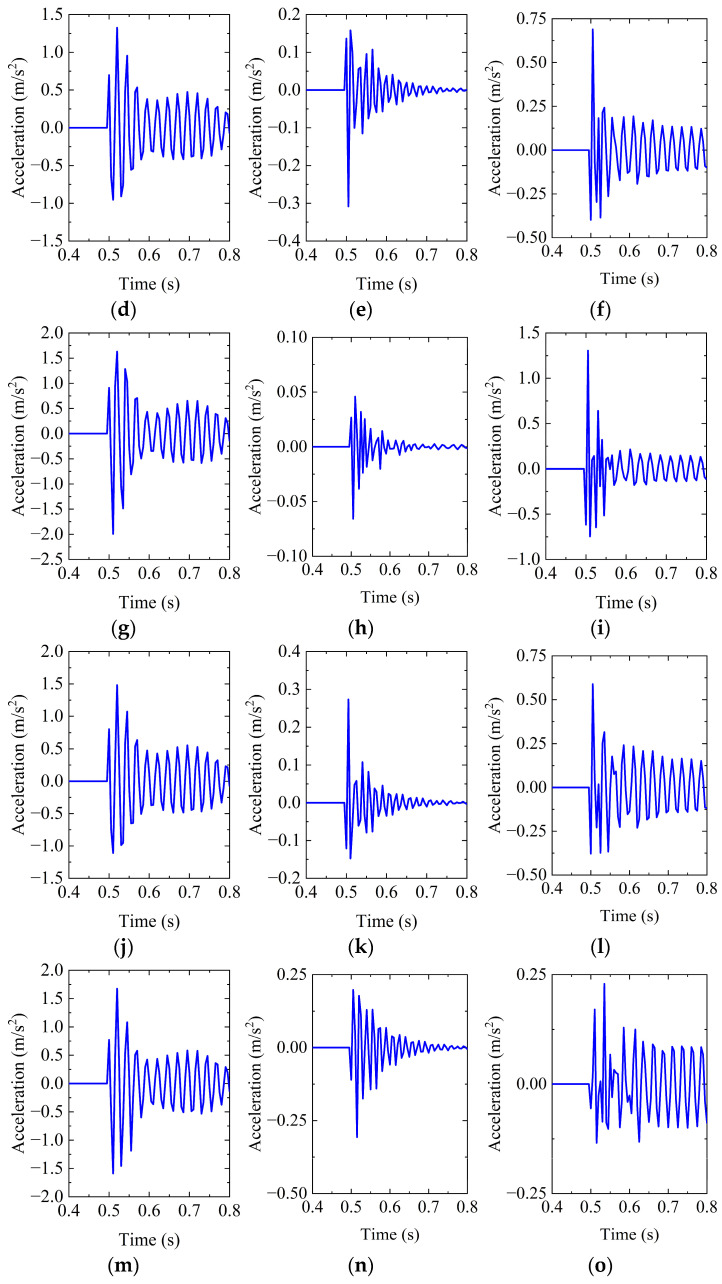
Structural responses under different load positions: (**a**) MX(L1-1000); (**b**) MY(L1-1000); (**c**) MZ(L1-1000); (**d**) MX(L2-1000); (**e**) MY(L2-1000); (**f**) MZ(L2-1000); (**g**) MX(L3-1000); (**h**) MY(L3-1000); (**i**) MZ(L3-1000); (**j**) MX(L4-1000); (**k**) MY(L4-1000); (**l**) MZ(L4-1000); (**m**) MX(L5-1000); (**n**) MY(L5-1000); and (**o**) MZ(L51000).

As shown in Figure 5, as the lateral load gradually approaches the monitoring point, axial vibration of the structure weakens, vertical vibration intensifies, while lateral vibration varies relatively little. This is reflected in the decrease in the peak acceleration in the Y direction, the increase in the peak acceleration in the Z direction, and the relative stability of the peak acceleration in the X direction. As shown in Figure 5b,n, the lateral loads under the two cases are positioned on opposite sides of the monitoring point, with similar distances to the point. The Y-direction components of the loads acting on the monitoring point are opposite, resulting in opposite acceleration responses in the Y direction. A similar pattern is observed in Figure 5e,k.

After applying impact loads at different locations, the acceleration responses at the monitoring point along the X, Y, and Z axes exhibit distinct characteristics, including variations in peak values, shifts in the dominant vibration direction, and differences in positive and negative amplitudes. These variations reflect inconsistencies in force transmission paths, axial propagation behavior, and local bending of the pipeline under different loading cases. Consequently, the three-axis acceleration measurements at the monitoring point contain information about both the position and direction of the applied impact. By comprehensively analyzing these acceleration responses, it is possible to infer the impact location and orientation, thereby enabling identification and localization of abnormal impact events along the pipeline.

## 4. Intelligent Multi-Parameter Sensor

### 4.1. Sensor Hardware Composition and Parameters

**Figure 6:**
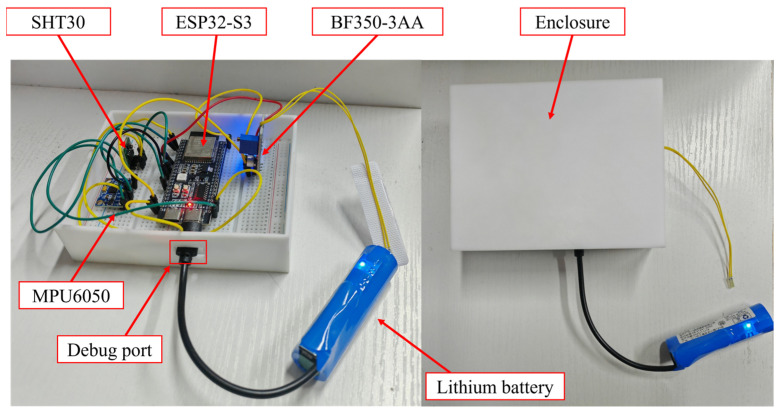
Sensor composition and enclosure.

As shown in Figure 6, the intelligent multi-parameter sensor developed in this study employs the ESP32-S3 as its central control unit and interfaces with the MPU6050 inertial measurement module, the SHT30 temperature and humidity module, and the BF350-3AA strain gauge module via I^2^C, UART, and ADC, respectively. This configuration enables the collaborative acquisition of three-axis acceleration, three-axis angular velocity, temperature, humidity, and strain data. The sensor housing is 3D-printed using a photosensitive resin, and power is supplied by a 5 V lithium battery module.

To facilitate strain measurement, a lead-out hole for the strain gauge is provided on the side of the housing, allowing the sensor’s internal strain gauge wiring to extend to the surface of the measured structure. The bottom of the housing includes a debugging port, through which a PC can connect to the ESP32-S3 for firmware programming, debugging, and serial communication. Due to the relatively large size of the current battery module, it is placed externally; however, the housing is designed with sufficient internal space, allowing a smaller lithium battery module to be fully integrated in future versions. The housing consists of an upper and lower half, forming a rectangular prism when assembled. For the prototype, the housing is sealed with tape to simplify installation. Future iterations may adopt a snap-fit design to allow easier opening, closing, and replacement of internal components.

The specific parameters of each functional module are as follows:

ESP32-S3: This controller integrates dual-mode wireless communication capabilities (Wi-Fi and Bluetooth LE), offers rich peripheral interfaces, and operates with a maximum CPU clock frequency of 240 MHz, supporting low-power operation and edge data processing.

MPU6050 inertial measurement module: This module integrates a three-axis accelerometer and a three-axis gyroscope. The selectable acceleration ranges are ±2 g, ±4 g, ±8 g, and ±16 g, while the angular velocity ranges are ±250°/s, ±500°/s, ±1000°/s, and ±2000°/s. The module supports a maximum sampling frequency of 1 kHz.

SHT30 temperature and humidity module: This module measures environmental temperature and humidity. Its temperature range is −40 °C to 125 °C with an accuracy of ±0.3 °C, and its humidity range is 0–100% RH with an accuracy of ±3%. The module supports periodic measurement mode, with a maximum reporting frequency of 10 Hz.

BF350-3AA strain gauge module: This module employs 350 metal foil strain gauges with a gauge factor of approximately 2.0 and an operating temperature range of −30 °C to 60 °C, suitable for monitoring elastic strain in general engineering structures.

### 4.2. Sensor Functions and Data Acquisition Display

The sensor implements edge computing algorithms on the ESP32-S3, including data preprocessing, feature extraction, rapid anomaly detection, and lightweight frequency-domain analysis. Upon startup, the sensor automatically collects a segment of acceleration and angular velocity data in a stationary state to compute the mean and standard deviation for each channel. These statistics are then used to build a zero-offset compensation model, mitigating sensor drift and enhancing the stability of subsequent measurements.

For time-domain analysis, using X-direction acceleration as an example, the sensor computes five real-time features: peak value, mean, variance, energy, and kurtosis. If the X-direction acceleration deviates from the stationary mean by more than three standard deviations, the sensor flags the current state as abnormal, enabling rapid early warning. In the frequency domain, the sensor incorporates a built-in 64-point fast Fourier transform (FFT) for lightweight spectral analysis.

**Figure 7:**
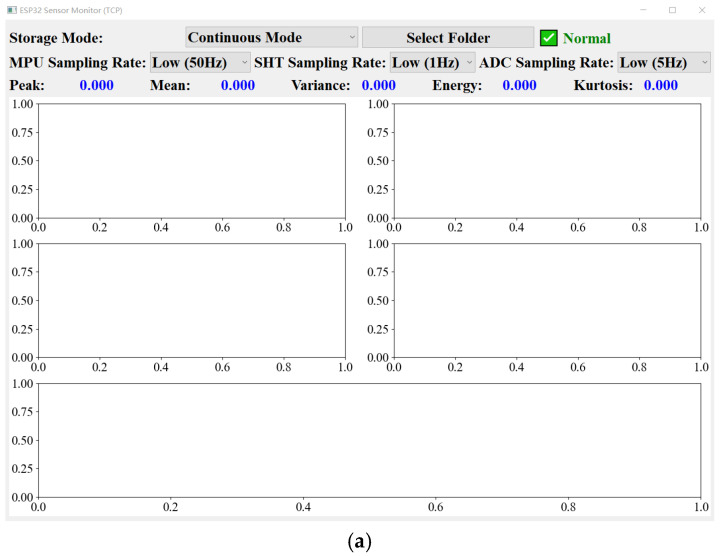

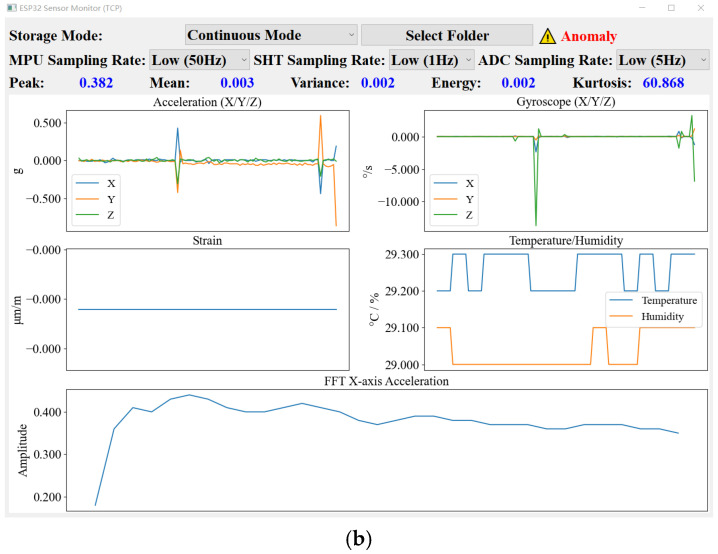
Sensor acquisition display: (**a**) initial display and (**b**) display showing event detected by sensor.

The sensor communicates with the PC via TCP over Wi-Fi and supports two operating modes: continuous acquisition and event-based acquisition. In the event-based mode, data is transmitted only when an anomaly is detected, reducing data transfer and conserving energy. As shown in Figure 7, dedicated acquisition software has been developed for the PC, enabling real-time display of multi-parameter sensor data, including three-axis acceleration, three-axis angular velocity, temperature, humidity, and strain. The software also provides functionality for selecting the acquisition mode, setting the data storage path, and adjusting the sampling frequency, allowing flexible switching between high-precision collection and low-power operation.

## 5. Laboratory Experiments and Method Validation

### 5.1. Experimental Design

**Figure 8:**
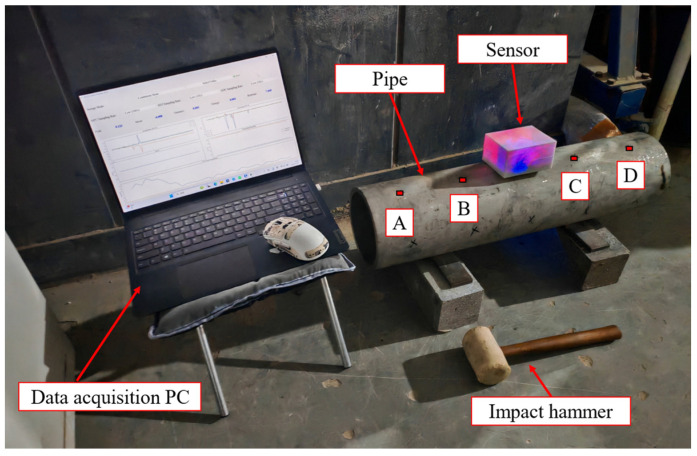
Experimental setup of the pipe segment.

As shown in Figure 8, to evaluate the monitoring performance of the developed intelligent multi-parameter sensor and to acquire experimental data for training the algorithm, a laboratory impact test was conducted on a GIL pipe segment. A 0.7 m-long stainless steel non-standard pipe section was used, with other geometric dimensions scaled down proportionally. Steel plates were welded to both ends of the pipe section to provide boundary constraints under impact. Four impact points were arranged along the top of the pipe section, labeled A, B, C, and D (see Figure 8). During the test, the impact hammer was repeatedly applied at each point in multiple rounds to simulate local impact loads the pipe segment might experience. The sensor was mounted at the midpoint on the top surface of the pipe section and firmly bonded to the pipe wall. Three-axis acceleration and three-axis angular velocity signals were simultaneously collected and transmitted in real time via Wi-Fi to the PC for visualization and data storage.

### 5.2. Experimental Results

#### 5.2.1. Original Sensor Data

**Figure 9:**
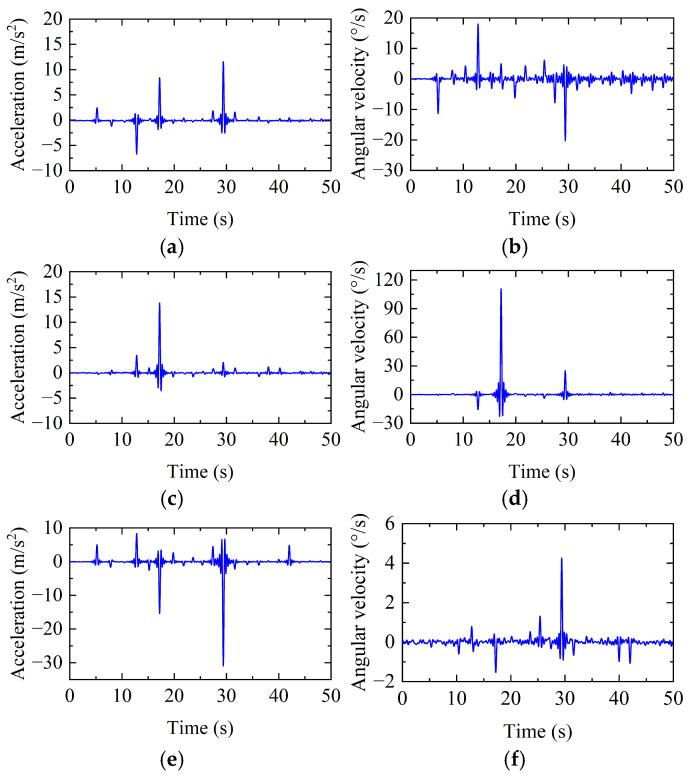
Triaxial acceleration and triaxial angular velocity responses under repeated impact events: (**a**) X-direction acceleration; (**b**) X-direction angular velocity; (**c**) Y-direction acceleration; (**d**) Y-direction angular velocity; (**e**) Z-direction acceleration; and (**f**) Z-direction angular velocity.

Figure 9 presents the triaxial acceleration and triaxial angular velocity responses recorded by the sensor during the first 50 s of the experiment. Each hammer strike produces a sharp transient change in acceleration, demonstrating that the developed sensor is capable of capturing the structural dynamic response and detecting high-frequency, abrupt signals induced by external impacts.

Meanwhile, the test results indicate that at the fixed monitoring point, both the triaxial acceleration and triaxial angular velocity vary depending on the impact position, exhibiting distinguishable patterns. Compared with the acceleration signals, the angular velocity signals generally display more pronounced fluctuations. This observation is consistent with the trends obtained from numerical simulations based solely on acceleration responses, suggesting that under impact loading, leveraging the multi-dimensional features formed by both triaxial acceleration and triaxial angular velocity facilitates more effective identification of the impact location.

#### 5.2.2. Algorithm Training and Testing

For the continuously recorded structural acceleration and angular velocity responses, the original data are segmented into individual impact events based on the peak characteristics of the signals. For each loading case, 80 valid impact segments are extracted for subsequent feature construction and model training. To construct the feature set, the three-axis acceleration and three-axis angular velocity signals are concatenated to provide a more comprehensive representation of the structural response induced by impact. An XGBoost multiclass classification model is used to identify the impact location, with the dataset split into a 4:1 ratio for training and testing. After the model is trained on the training set, its performance is evaluated on the independent test set.

**Figure 10:**
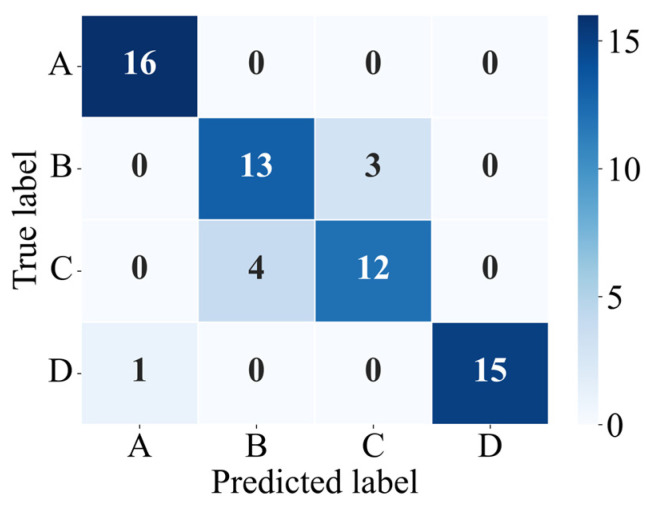
Confusion matrix of the test set.

To evaluate the classification performance of the model, the confusion matrix for the test set is plotted, as shown in Figure 10. The confusion matrix provides a visual summary of the model’s prediction outcomes across different classes: each row corresponds to the true class, each column to the predicted class, diagonal entries indicate correctly classified samples, and off-diagonal entries reflect misclassifications between classes [38]. Based on the confusion matrix, the Precision, Recall, and F1-score metrics for each impact location are further computed, as reported in Table 3. Here, Precision denotes the proportion of samples predicted as a given class that truly belong to that class; Recall represents the proportion of samples that truly belong to a given class and are correctly identified; and the F1-score is the harmonic mean of Precision and Recall, calculated according to Equations (2)–(4) [39]. Overall, the model demonstrates strong recognition performance across all four impact cases, achieving an average classification accuracy of 0.8750.

(2) $${\mathrm{Precision}}_{i}=\frac{{\mathrm{TP}}_{i}}{{\mathrm{TP}}_{i}+{\mathrm{FP}}_{i}}$$

(3) $${\mathrm{Recall}}_{i}=\frac{{\mathrm{TP}}_{i}}{{\mathrm{TP}}_{i}+{\mathrm{FN}}_{i}}$$

(4) $${F1\text{-}\mathrm{score}}_{i}=2\times \frac{{\mathrm{Precision}}_{i}\times {\mathrm{Recall}}_{i}}{{\mathrm{Precision}}_{i}+{\mathrm{Recall}}_{i}}$$

where ${\mathrm{TP}}_{i}$ denotes the number of samples of the i-th class that are correctly predicted, ${\mathrm{FP}}_{i}$ denotes the number of samples incorrectly predicted as the i-th class, and ${\mathrm{FN}}_{i}$ denotes the number of samples that truly belong to the i-th class but are misclassified.

In addition to conventional classification performance metrics, the mean distance error (MDE) is introduced to further quantify the spatial deviation between the predicted and true impact locations. Taking the monitoring point as the coordinate origin, the coordinate of point A ($x_{A}$) is −0.28 m, that of point B ($x_{B}$) is −0.14 m, that of point C ($x_{C}$) is 0.14 m, and that of point D ($x_{D}$) is 0.28 m. Let the true label of the i-th test sample be $y_{i}\in \left\{A,B,C,D\right\}$, the predicted label be ${\hat{y}}_{i}\in \left\{A,B,C,D\right\}$, and the total number of test samples be N. The calculation of the mean distance error is given in Equation (5). A smaller MDE indicates a smaller spatial discrepancy between the predicted and true impact locations, reflecting stronger localization capability of the model. The MDE computed from the confusion matrix is 0.0394 m, indicating that the model’s identification of impact locations is highly consistent with the true positions.

To comprehensively assess the model’s classification consistency, the Kappa coefficient is calculated based on the confusion matrix, with the computation formulas shown in Equations (6)–(8). The resulting Kappa coefficient is 0.8333, indicating that the model demonstrates high classification consistency and strong reliability in identifying impact positions.

(5) $$\mathrm{MDE}=\frac{1}{N}\sum_{i=1}^{N}\left|x_{{\hat{y}}_{i}}-x_{y_{i}}\right|$$

(6) $$K=\frac{P_{o}-P_{e}}{1-P_{e}}$$

(7) $$P_{o}=\frac{\sum_{i}n_{ii}}{N}$$

(8) $$P_{e}=\frac{1}{N^{2}}\sum_{i}n_{i\cdot }n_{\cdot i}$$

where $n_{ii}$ represents the diagonal element of the confusion matrix, $n_{i\cdot }$ and $n_{\cdot i}$ are the sums of the i-th row and i-th column, respectively, and N denotes the total number of samples.

#### 5.2.3. Impact of Feature Dimensionality

**Figure 11:**
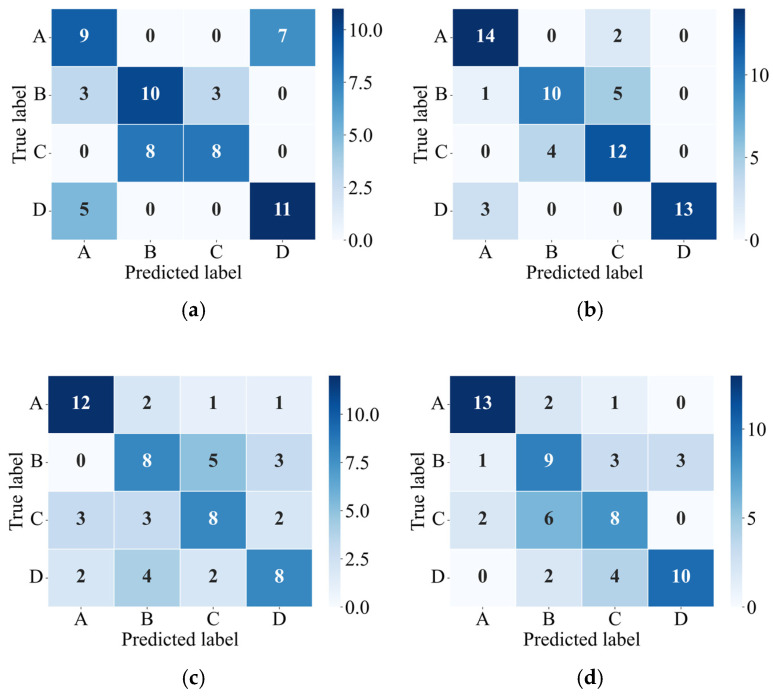
Confusion matrices of the test set under different feature inputs: (**a**) X-direction acceleration; (**b**) triaxial acceleration; (**c**) X-direction angular velocity; and (**d**) triaxial angular velocity.

To investigate the influence of different physical quantities on impact position recognition performance, this study compares four feature input strategies: using only X-direction acceleration, only triaxial acceleration, only X-direction angular velocity, and only triaxial angular velocity. Each feature set is used to train and test the XGBoost model independently. The confusion matrices for the test set are shown in Figure 11. The average recognition accuracies are 0.5938, 0.7656, 0.5625, and 0.6250, with corresponding Kappa coefficients of 0.4583, 0.6875, 0.4167, and 0.5000. The MDE for the four feature strategies are 0.1597 m, 0.0810 m, 0.1466 m, and 0.1072 m, respectively. The results indicate that coupled responses of acceleration and angular velocity across different directions are critical for distinguishing impact positions, with acceleration features contributing more than angular velocity. Recognition performance using any single-type feature is lower than that achieved by combining three-axis acceleration and angular velocity, demonstrating that multi-source features provide a more comprehensive representation of structural dynamic responses and improve impact position discrimination.

#### 5.2.4. Model Comparison and Selection Analysis

**Figure 12:**
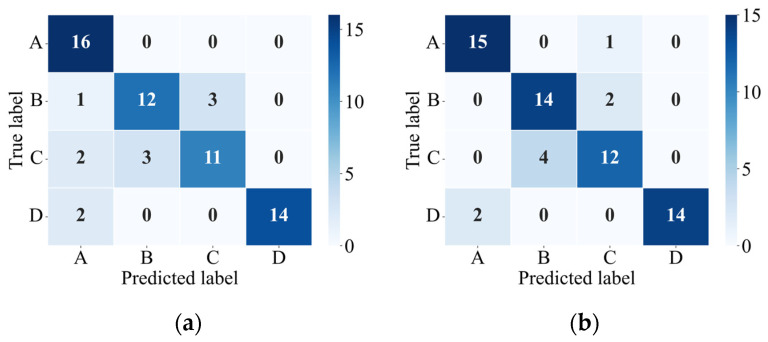
Confusion matrices of the test set for different models: (**a**) SVM and (**b**) Random Forest.

To verify the rationality of the model selection, XGBoost, support vector machine (SVM), and Random Forest are compared using the same dataset. All three models adopt an identical training-test split strategy. The confusion matrices for SVM and Random Forest are presented in Figure 12, while those for XGBoost is shown in Figure 10. Table 4 summarizes the average accuracy, Kappa coefficient, and mean distance error for the three models. The results indicate that XGBoost outperforms SVM and Random Forest across all evaluation metrics, achieving higher classification accuracy, stronger classification consistency, and superior localization performance. These findings demonstrate that XGBoost is more suitable for the multi-class impact location recognition task addressed in this study.

#### 5.2.5. Robustness and Generalization Analysis

**Figure 13:**
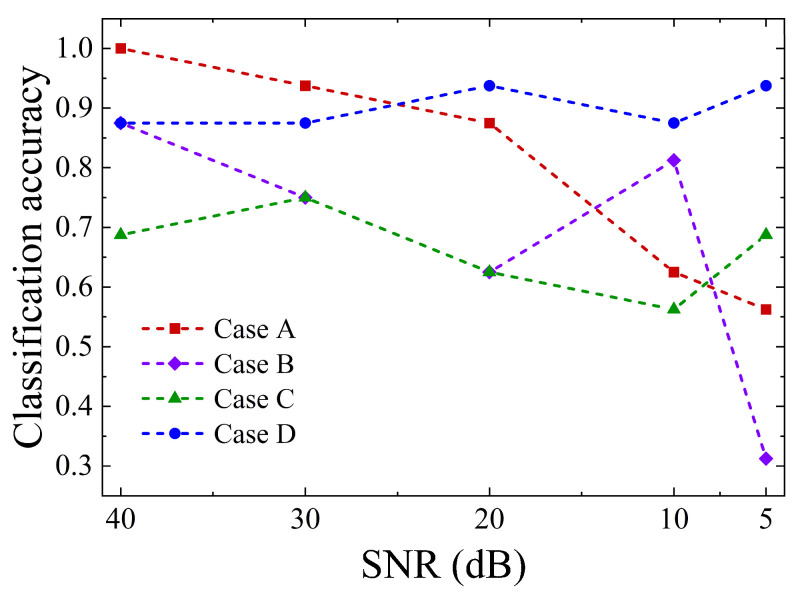
Classification accuracy of each case under different noise levels.

In real GIL pipeline operating environments, sensor measurements can be affected by various sources of interference, including mechanical noise, electromagnetic coupling, temperature drift, and power supply fluctuations. To evaluate the robustness of XGBoost for impact location identification, Gaussian white noise of varying intensities is added to the acceleration and angular velocity signals during testing, simulating five typical scenarios with signal-to-noise ratios (SNR) of 40 dB, 30 dB, 20 dB, 10 dB, and 5 dB, representing field disturbances from mild to severe. Table 5 summarizes the model’s average accuracy, Kappa coefficient, and mean distance error under different noise levels, while Figure 13 illustrates the variation in classification accuracy for each case. At an SNR of 20 dB, the model achieves an average accuracy of 0.7656, a Kappa coefficient of 0.6875, and a mean distance error of 0.0766 m. Even at the extremely low SNR of 5 dB, the model maintains an average accuracy of 0.6250. These results demonstrate the model’s strong robustness against moderate- to low-intensity noise. Compared with cases A and B, cases C and D show smaller variations in classification accuracy across different noise levels, indicating they are less sensitive to noise. Overall, the XGBoost approach adopted in this study exhibits high practical applicability and potential for deployment in real-world GIL pipeline monitoring scenarios.

**Figure 14:**
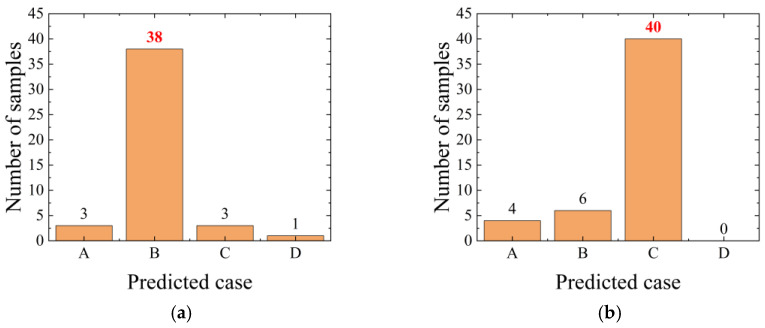
Predicted impact positions for new test points in previously unseen locations: (**a**) midpoint E of AB interval and (**b**) midpoint F of CD interval.

In Section 5.2.2, the XGBoost model is trained exclusively on impact loads at four fixed positions, A, B, C, and D. To assess the model’s generalization to unseen impact locations, a new test point E (coordinate $x_{E}$ = −0.21 m) is added at the midpoint of the AB interval, and a new test point F (coordinate $x_{F}$ = 0.21 m) is added at the midpoint of the CD interval. Fifty impacts are applied at each of these new positions to form a supplementary test set. These points are not included in the training process and are used solely to evaluate the model’s extrapolation performance in continuous space. The new test data are fed into the XGBoost model trained on only the four original cases (A, B, C, and D). The recognition results are shown in Figure 14. At point E, the model most frequently predicts position B, with 38 occurrences, while at point F, it most frequently predicts position C, with 40 occurrences. These results indicate that, even without having learned the response patterns of the monitoring point to impacts at positions E and F, the model’s predictions cluster around the nearest known categories, demonstrating that it retains a certain degree of spatial generalization under limited discrete labels.

### 5.3. GIL Monitoring Cloud Platform

**Figure 15:**
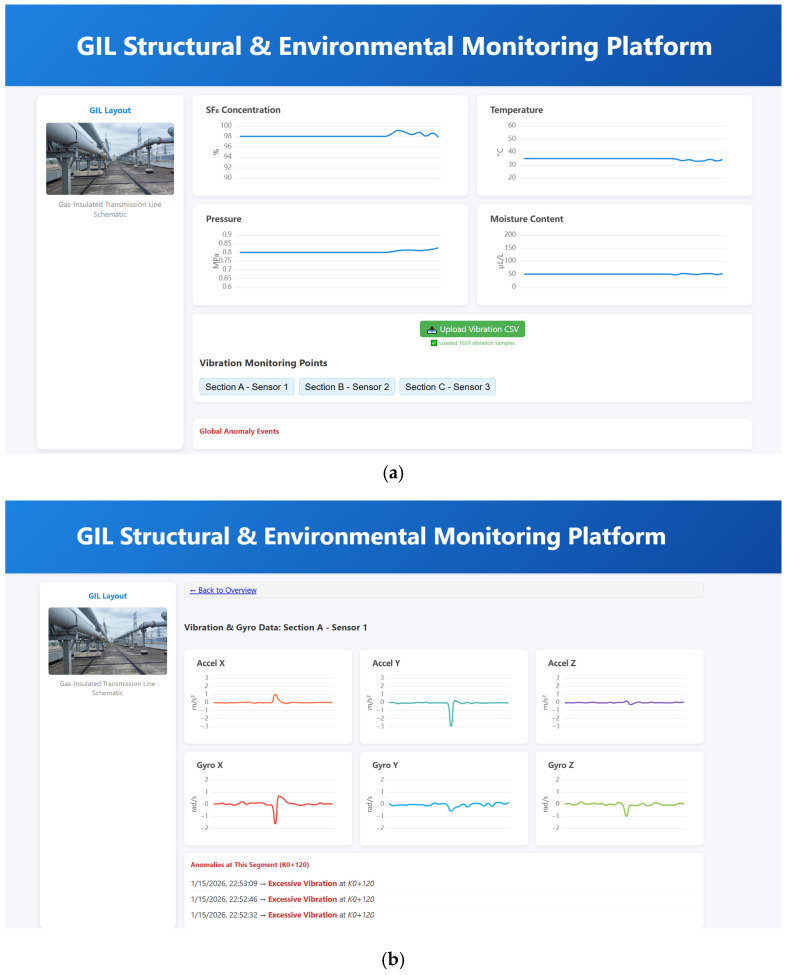
Lightweight cloud platform for GIL monitoring: (**a**) homepage displaying environmental parameters; (**b**) detailed view of sensor data.

As shown in Figure 15, a lightweight cloud platform is developed for monitoring the structural health and operational environment of GIL pipelines, enabling the PC to upload monitoring data to the cloud and manage historical records remotely. As shown in Figure 15a, the platform homepage displays trends of key environmental parameters during daily GIL operation, including SF_6_ concentration, temperature, pressure, and humidity. By clicking on a sensor identifier (e.g., ‘Section A-Sensor 1’), users can access a detailed view of the corresponding monitoring point to examine the triaxial acceleration and triaxial angular velocity data collected at that location (see Figure 15b).

As the cloud platform is primarily intended for displaying and comparing historical data, sensor original data and corresponding anomaly detection results are uploaded manually, without requiring real-time transmission. To reduce data transmission while preserving the core signal features, acceleration and angular velocity signals are moderately downsampled before upload. Once uploaded, the platform automatically scrolls through historical monitoring data and anomaly detection results, continuously recording them. This provides a unified data access point for personnel involved in operation, maintenance, monitoring, and management, facilitating traceability and comparative analysis of the GIL’s operational status.

### 5.4. Limitations

This paper develops a three-tier end-edge-cloud collaborative architecture for impact anomaly monitoring on GIL pipelines. Owing to experimental constraints, a non-standard pipe section model is constructed in the laboratory, whose geometric dimensions, support conditions, and material properties differ from those of pipe sections in real engineering applications. Such discrepancies may influence the structural dynamic response and limit the direct generalization of the experimental results to practical scenarios. However, the objective of this study is not to fully replicate real operating conditions, but rather to validate the functional effectiveness of the proposed intelligent multi-parameter sensor and to assess the feasibility of the XGBoost algorithm for impact position recognition under controlled conditions. Accordingly, the model simplifications are reasonable within the scope of this study, while the applicability of the proposed approach to complex real-world environments warrants further investigation.

Considering potential interference factors in real operating environments, such as temperature variations and electromagnetic coupling, this study adopts a simplified simulation approach by superimposing Gaussian white noise of varying intensities onto the original signals. This simplification cannot fully capture the nonstationary characteristics of noise in practical scenarios and only represents its effects on the signals to a limited extent. Experimental results indicate that, under moderate and low noise levels, the recognition performance of the proposed method does not exhibit significant degradation; however, its robustness under complex real-world interference conditions requires further investigation.

In addition, this study completes the preliminary development of a cloud platform, which currently supports manual data uploading as well as basic data statistics and visualization functions. Although the platform still has limitations in terms of user interaction design and functional completeness, it successfully achieves data transmission and visualization from the PC to the cloud, thereby validating the technical feasibility of the proposed end-edge-cloud architecture.

Overall, this study primarily focuses on the development and validation of the proposed monitoring methodology and system architecture. The experimental results provide an initial verification of the feasibility of the approach; however, its performance, stability, and applicability under real engineering conditions remain to be systematically evaluated through future field deployments and long-term operational testing.

## 6. Conclusions

This paper presents a three-tier end-edge-cloud monitoring system for detecting impact anomalies on GIL pipelines. The intelligent multi-parameter sensor is developed, and an impact position recognition algorithm is trained on the PC using laboratory test data. A cloud-based platform is established for structural health and environmental monitoring. The main conclusions are as follows.

1. Numerical simulation results show that when impact loads are applied at different positions on the GIL pipeline, the acceleration responses at the monitoring point located at the middle of the pipe section exhibit distinct characteristics in the X, Y, and Z directions, including variations in peak values, shifts in the main vibration direction, and differences between positive and negative responses. A comprehensive analysis of the multi-dimensional dynamic response at the monitoring point allows for effective inference of the impact load’s position and direction, providing a basis for optimizing sensor placement and reducing the number of monitoring points in practical engineering applications.
2. The developed intelligent multi-parameter sensor can simultaneously acquire multiple physical quantities, including three-axis acceleration, three-axis angular velocity, temperature, humidity, and strain. It also executes lightweight data-processing algorithms locally, performing tasks such as noise reduction, time- and frequency-domain feature extraction, and rapid threshold-based anomaly detection.
3. The training results of the XGBoost multi-classification model indicate that the coupled responses of acceleration and angular velocity in different directions play a crucial role in distinguishing impact positions, with acceleration contributing more significantly. A six-dimensional feature set combining triaxial acceleration and triaxial angular velocity achieves optimal classification performance. The model attains an average recognition accuracy of 0.8750 and a Kappa coefficient of 0.8333, demonstrating high classification consistency and reliability. These results suggest that multi-source feature fusion effectively captures the complex dynamic response of the structure under impact, thereby enhancing the model’s ability to discriminate impact positions.
4. The end-edge-cloud lightweight monitoring system developed in this study implements a complete monitoring chain, from multi-source data acquisition and rapid event detection to impact localization and cloud-based historical information management. It provides a cost-effective and scalable intelligent solution for assessing the structural health and operational environment of GIL pipelines.

Future work will focus on further optimizing the hardware integration and low-power performance of the intelligent multi-parameter sensor, as well as enhancing the data management and visualization capabilities of the cloud platform. In addition, long-term on-site operational data will be used to validate the performance of the monitoring system, improving its applicability and stability in real-world engineering environments.

## Figures and Tables

**Table 1:** Loading case names (top loading).

Section	Case Name		
	Load Peak: 500 N	Load Peak: 1000 N	Load Peak: 2000 N
1	T1-500	T1-1000	T1-2000
2	T2-500	T2-1000	T2-2000
4	T4-500	T4-1000	T4-2000
5	T5-500	T5-1000	T5-2000

**Table 2:** Loading case names (lateral loading).

Section	Case Name		
	Load Peak: 500 N	Load Peak: 1000 N	Load Peak: 2000 N
1	L1-500	L1-1000	L1-2000
2	L2-500	L2-1000	L2-2000
3	L3-500	L3-1000	L3-2000
4	L4-500	L4-1000	L4-2000
5	L5-500	L5-1000	L5-2000

**Table 3:** Classification metrics for different cases.

Case Name	Precision	Recall	F1-Score
A	0.9412	1.0000	0.9697
B	0.7647	0.8125	0.7879
C	0.8000	0.7500	0.7742
D	1.0000	0.9375	0.9677

**Table 4:** Performance comparison of different models.

Model	Average Accuracy	Kappa Coefficient	Mean Distance Error
SVM	0.8281	0.7708	0.0591 m
Random Forest	0.8594	0.8125	0.0503 m
XGBoost	0.8750	0.8333	0.0394 m

**Table 5:** Performance comparison under different noise levels.

SNR	Average Accuracy	Kappa Coefficient	Mean Distance Error
40 dB	0.8594	0.8125	0.0481 m
30 dB	0.8281	0.7708	0.0591 m
20 dB	0.7656	0.6875	0.0766 m
10 dB	0.7188	0.6250	0.1116 m
5 dB	0.6250	0.5000	0.1356 m

## Data Availability

Data will be made available on request.

## Abbreviations

The following abbreviations are used in this manuscript:

GIL	Gas-insulated transmission lines
XGBoost	Extreme gradient boosting
PD	Partial discharge
UHF	Ultra-high-frequency
TDoA	Time-difference-of-arrival
CCF	Cross-correlation function
ME	Minimum energy
TV	Threshold value
RSS	Received signal strength
PDIV	PD inception voltage
CEEMD	Complementary ensemble empirical mode decomposition
WOA-ELM	Whale optimization extreme learning machine
PCA	Principal component analysis
SVM	Support vector machine
SOA	Seagull optimization algorithm
DAS	Distributed acoustic sensing
AE	Acoustic emission
PC	Personal computer
GBDT	Gradient boosting decision tree
FFT	Fast Fourier transform
MDE	Mean distance error
SNR	Signal-to-noise ratio
